# Supplementary material for: Analysis of PPARγ Signaling Activity in Psoriasis
Source: Int J Mol Sci. 2021 Aug 10;22(16):8603. doi: 10.3390/ijms22168603 (PMC8395241; doi:10.3390/ijms22168603)
Supplement: Supplementary file 1 [file ijms-22-08603-s001.zip › Supplemental materials_Analysis of PPARg signaling activity in psoriasis/Pathway models/Models images and html files/PPARG inhibits phenotypes assotiated with psoriasis/125626.html]

pneumonia


# Disease pneumonia

|  |  |
| --- | --- |
| URN | urn:agi-meshdis:Pneumonia |
| Total Entities | 26 |
| Connectivity | 5779 |
| Name | pneumonia |
| ObjectType | Disease |

---

|  |  |
| --- | --- |
| ChildConcepts | interstitial pneumonia |
|  | infectious pneumonia |
|  | acute chest syndrome |
|  | Escherichia coli pneumonia |
|  | hospital-acquired pneumonia |
|  | pulmonary infiltrate |
|  | organizing pneumonia |
|  | Pasteurella multocida pneumonia |
|  | Pseudomonas aeruginosa pneumonia |
|  | healthcare-associated pneumonia |
|  | experimental pneumonia |
|  | neonatal pneumonia |
|  | necrotizing pneumonia |
|  | acute berylliosis |
|  | cholesterol pneumonia |
|  | hypersensitivity pneumonitis |
|  | bronchopneumonia |
|  | ventilator-associated pneumonia |
|  | pleuropneumonia |
|  | aspiration pneumonia |
|  | fog fever |
|  | maedi |
|  | rickettsial pneumonia |
|  | mycoplasmal pneumonia of swine |
|  | enzootic pneumonia of calves |
|  | Staphylococcal necrotizing pneumonia |

---

|  |  |
| --- | --- |
| Pathway | PPARG inhibits phenotypes assotiated with psoriasis |
|  | Neighbors of pneumonia |

---

|  |  |
| --- | --- |
| MedScan ID | 9003840 |

---

|  |  |
| --- | --- |
| Alias | superficial pneumonia |
|  | Pulmonary Inflammation |
|  | mouse pneumonitis |
|  | Lung Inflammations |
|  | nosocomial pneumonia |
|  | acute diffuse pneumonias |
|  | pneumonia superficialis |
|  | Lung Inflammation |
|  | pneumonitis |
|  | pneumonic lung |
|  | nosocomial pneumonias |
|  | acute pneumonitis |
|  | Inflammations, Lung |
|  | enzootic pneumonia |
|  | non specific inflammatory lung diseases |
|  | Pneumonias |
|  | Inflammation, Lung |
|  | Pneumonia |
|  | inflammatory disease of the lung |
|  | atrophic reticular pneumonia |
|  | non specific inflammatory lung disease |
|  | pulmonal inflammation |
|  | nonspecific inflammatory lung diseases |
|  | Inflammation, Pulmonary |
|  | inflammatory lung disease |
|  | pulmonic inflammation |
|  | inflammatory diseases of the lung |
|  | Pulmonary Inflammations |
|  | inflammatory lung diseases |
|  | pulmonal inflammations |
|  | pneumonitis acuta |
|  | nonspecific inflammatory lung disease |
|  | lobitis |
|  | acute diffuse pneumonia |
|  | pneumonic lungs |
|  | Inflammations, Pulmonary |
|  | stable pneumonia |
|  | peripneumonia |
|  | Pneumonitides |

---

|  |  |
| --- | --- |
| MeSH ID | D011014 |
|  | M0017064 |

---

|  |  |
| --- | --- |
| MeSH Heading | Pneumonia |

---
